# Causal association of circulating cholesterol levels with dementia: a mendelian randomization meta-analysis

**DOI:** 10.1038/s41398-020-0822-x

**Published:** 2020-05-12

**Authors:** Xiaoyu Zhang, Qiuyue Tian, Di Liu, Tao Geng, Xizhu Xu, Siqi Ge, Deqiang Zheng, Lijuan Wu, Manshu Song, Haifeng Hou, Wei Wang, Youxin Wang

**Affiliations:** 1 Department of Epidemiology and Health Statistics, School of Public Health, Beijing Municipal Key Laboratory of Clinical Epidemiology, Capital Medical University, Beijing, 100069 China; 2 Geriatric Department, Emergency General Hospital, Beijing, 100028 China; 3 School of Public Health, Shandong First Medical University & Shandong Academy of Medical Sciences, Tai’an, 271000 China; 4 Beijing Neurosurgical Institute, Capital Medical University, Beijing, 100070 China; 5 School of Medical and Health Sciences, Edith Cowan University, Perth, WA 6027 Australia

**Keywords:** Diagnostic markers, Clinical genetics

## Abstract

Prospective studies have shown that abnormally circulating cholesterol is associated with the risk of dementia. However, whether the association is causal or not remains unclear. We attempt to infer the causal association in a MR meta-analysis by using *ApoE* gene polymorphisms as instrument variables. Studies with dementia risk (27 studies) or circulating lipid levels (7 studies) were included, with totally 3136 dementia patients and 3103 healthy controls. The analyses showed that carriers of ε2 allele significantly were of decreased risk of AD (OR = 0.70; 95% CI: 0.58–0.84; *P* < 0.01), whereas carriers of ε4 allele were of increased risk of AD (OR = 3.62; 95% CI: 3.03–4.32; *P* < 0.05), compared to these of ε3 allele. Circulating TC was significantly reduced in carriers of ε2 allele (WMD = − 0.29 mmol/L; 95% CI: −0.54 to −0.03; *P* < 0.05) and increased in carriers of ε4 allele (WMD = 0.42 mmol/l; 95% CI: 0.001–0.84; *P* < 0.05). In addition, carriers of ε4 allele had reduction in circulating HDL-C (WMD = − 0.04 mmol/L; 95% CI: − 0.07 to −0.001; *P* < 0.05). In comparing allele ε2 with ε3, the predicted OR of having AD for 1 mg/dL increment in circulating TC was 0.97 (95% CI: 0.86–0.98; *P* < 0.05). Comparing allele ε4 with ε3, the predicted OR for a 1 mg/dL increment in TC was 1.08 (95% CI: 1.05–17.58; *P* < 0.05), and reduction in HDL-C was 2.30 (95% CI: 1.51–43.99; *P* < 0.05). Our findings demonstrate that high circulating TC and reduced HDL-C levels might be potential risk factors of the development of AD.

## Introduction

Dementia is a syndrome caused by a variety of brain illnesses that have adverse impact on memory, thinking, behavior and the ability to perform daily activities. Alzheimer’s disease (AD) and vascular dementia (VaD) account for more than 90% of the dementia cases^1^. The number of dementia patients currently is 47 million and estimated to 75 million by 2030 in globe^2^. The increasing burden of dementia emphasizes the necessity to identify risk factors underlying prevention of dementia.

Some prospective studies have revealed that circulating lipid abnormalities are independent indicators of the development of dementia^3–7^. In addition, dyslipidemia is a risk factor for vascular diseases, which is clearly associated with VaD and AD^8^. High total cholesterol (TC) levels were found to be positively associated with the risk of AD in a dose–response manner^9^, while higher high-density lipoprotein cholesterol (HDL-C) (>55 mg/dL) resulted in decreased risk of AD^10^. However, confounding factors such as age and sex may also explain some of the observed associations, suggesting that the value is susceptible to confounding.

Differences in the amino acid sequence of apolipoprotein E (ApoE) are major determinants of plasma cholesterol levels within a population level. ApoE has a key role in the clearance of cholesterol from plasma^11^. It has been estimated that nearly 60% of circulating cholesterol variation is under genetic control and thereout 14% variation is ascribed to *ApoE* genetic defects^12^. The synthesis of ApoE is controlled by three independent alleles such as ε2, ε3 and ε4, corresponding to 6 *ApoE* genotypes (ε2ε2, ε3ε2, ε4ε2, ε3ε3, ε4ε3 and ε4ε4)^13^. Genotypes ε4/ε4 and ε4/ε3 are associated with high cholesterol concentration in blood^14–17^. In addition, polymorphisms of the *ApoE* gene have been studied extensively in the context of a variety of clinical endpoints such as hypercholesterolemia^18,19^, ischemic heart disease^20,21^, stroke^22^ and hypertension^23,24^.

In the absence of trials, genetic studies can be used to help evaluate causality. This approach is known as Mendelian randomization (MR) which can be used to assess gene-related risk factors for causal associations with clinical outcomes. MR analyses are based on Mendel’s observation that inheritance of one trait should be independent of the inheritance of other traits^25^. To test this hypothesis and provide an unbiased estimation of the causality, MR meta-analysis was used to assess the causality between circulating lipid profiles and the risk of dementia.

## Materials and Methods

The present meta-analysis was undertaken in accordance with the guidelines formulated in the Preferred Reporting Items for Systematic Reviews and Meta-analyses (PRISMA) statement^26^.

### Search strategy for qualified studies

To identify all relevant articles that addressed the associations of *ApoE* gene ε2/ε3/ε4 polymorphism with dementia or circulating lipid changes, we systematically searched PubMed and the Excerpta Medica database (EMBASE) for literature in the English language. For the research strategy, logic based on specific descriptors was adopted (English language) in combination with Boolean operators (and/or), with the aid of parentheses to define intercalation within the same logic and the quotation marks to identify the compound words. The search was conducted using the following search terms: (APOE, “Apolipoprotein E” or “apo-E”) and (polymorphism, allele, variant, variation, genotype, mutation, SNP or isoforms) and (dementia, “Alzheimer*” or “vascular dementia”) and (“lipid profile”, “total cholesterol”, triglyceride, triacylglycerol, “high-density lipoprotein cholesterol”, “low-density lipoprotein cholesterol”, TC, “HDL-C”, “LDL-C” or TG). All selected articles were written in English and published before 20 June 2019. In addition, we manually scrutinized the reference list of eligible literature.

The titles and abstracts of all retrieved articles were read independently by two authors (Xiaoyu Zhang and Qiuyue Tian). For articles that could not be ascertained, the full text and supplementary data were reviewed. The process was conducted independently by the two authors. The discrepancy was adjudicated by a discussion with the third author (Di Liu).

### Inclusion and exclusion criteria

The literatures that met the following criteria were included in the meta-analysis: 1) detailed allele or genotype counts of *ApoE* ε2/ε3/ε4 gene polymorphism should be available between dementia patients and controls in case-control studies (including nested case-control studies); 2) the mean or median values (standard deviations) on TC, HDL-C, LDL-C and triglyceride (TG) levels were found across *ApoE* gene ε2/ε3/ε4 alleles or genotypes in case-control (including nested case-control) or cohort studies.

The exclusion criteria were as follows: 1) studies that examined the progression, severity or response to treatment of dementia in association with *ApoE* gene ε2/ε3/ε4 polymorphism or a lack of healthy controls; 2) case reports or series, editorials, narrative or systematic reviews, conference abstracts or proceedings and non-English articles.

### Literature quality assessment

The Newcastle–Ottawa Scale was used to assess the methodological quality of the included studies^27^. For case-control studies, this comprised the determination of (1) adequate case definition, (2) representativeness of cases, (3) selection of control, (4) definition of control, (5) comparability of case and control groups, (6) exposure, (7) whether there were identical exposure methods for cases and controls, and (8) non-response rate. For cohort studies, this comprised the determination of (1) representativeness of the exposed cohort, (2) selection of the unexposed cohort, (3) exposure, (4) whether the study subjects had an ending event that occurred before the study began, (5) comparability of the cohorts, (6) evaluation of the ending event, (7) whether follow-up was sufficient, and (8) integrity of follow-up examinations. Each item that met one of the above-mentioned criteria was represented by ∗, and each ∗ was equivalent to 1 point, giving a potential total of 9 points. Higher scores indicated higher quality studies. Moreover, studies with a score of 6 points and above were included in the present meta-analysis.

### Extracted information

Data including the first author’s last name, publication year, ethnicity, dementia subtype, sample size, allele counts of *ApoE* gene ε2/ε3/ε4 polymorphisms between dementia patients and controls, the mean or median (standard deviation) values of TC, TG, HDL-C and LDL-C levels across *ApoE* gene ε2/ε3/ε4 carriers were extracted independently from each qualified study by two investigators (Xiaoyu Zhang and Qiuyue Tian). The units of circulating TG, TC, HDL-C and LDL-C were consistently standardized to millimole per liter (mmol/L). The following data collection and article quality were assessed in duplicate.

### Statistical analyses

Data management and statistical analyses were performed with Stata software (StataCorp, TX, USA, version 12.0 for Windows). The odds ratios (ORs) and 95% confidence intervals (CIs) were calculated to express the distributional differences of alleles of *ApoE* gene ε2/ε3/ε4 polymorphism between patients with dementia and the controls. Weighted mean differences (WMDs) and 95% CIs were calculated to compare the changes in circulating levels of TC, TG, HDL-C and LDL-C across allele carriers.

In this meta-based MR analysis, if the *I*^2^ values were <50%, then the fixed effects model was selected to calculate the pooled ORs and 95% CI^28^. Otherwise, a random effects model was applied to combine effect-size estimates. Predetermined subgroup analyses were undertaken prior in terms of dementia subtype (AD and VaD), sample size, ethnicity or gender. The probability of publication bias was determined visually by Begg’s funnel plot and Egger’s regression asymmetry test.

Under the assumptions of MR, we calculated the risk prediction as a ratio of the coefficient for the association between *ApoE* gene ε2/ε3/ε4 polymorphism and dementia risk to that of the relationship between the polymorphism and circulating lipid changes. Statistical significance was considered when a two-tailed *P* value was less than 0.05.

## Results

### Eligibility criteria

The flow chart of the selection process was shown in Supplementary Fig. S1. For the association between *ApoE* gene polymorphism ε2/ε3/ε4 and dementia, there were 27 studies with 3136 dementia patients and 3103 healthy controls^29–41^. For the association between *ApoE* gene ε2/ε3/ε4 polymorphism and circulating cholesterol, five studies addressed HDL-C^35,42–45^, five studies addressed LDL-C^35,42–45^, seven studies addressed TG^35,42–47^ and six studies addressed TC^35,42–46^, were included.

### Association of the *ApoE* gene alleles with dementia

**Fig. 1:**
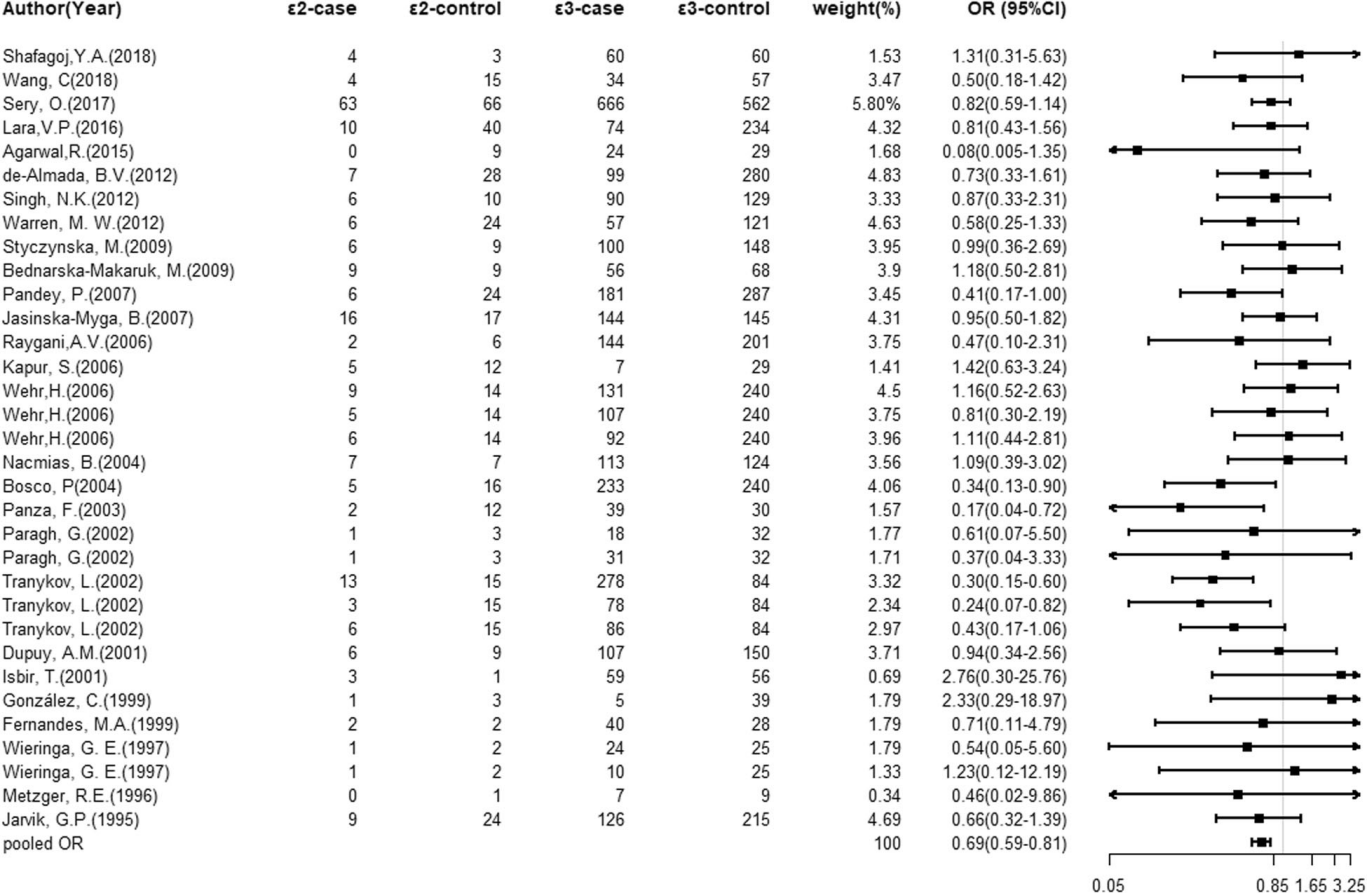
Overall comparisons of *ApoE* gene ε2 versus ε3 in association with dementia risk. Forest plots of the dementia risk associated with the *ApoE* gene ε2/ε3/ε4 alleles for ε2 vs. ε3 in all study populations.

**Fig. 2:**
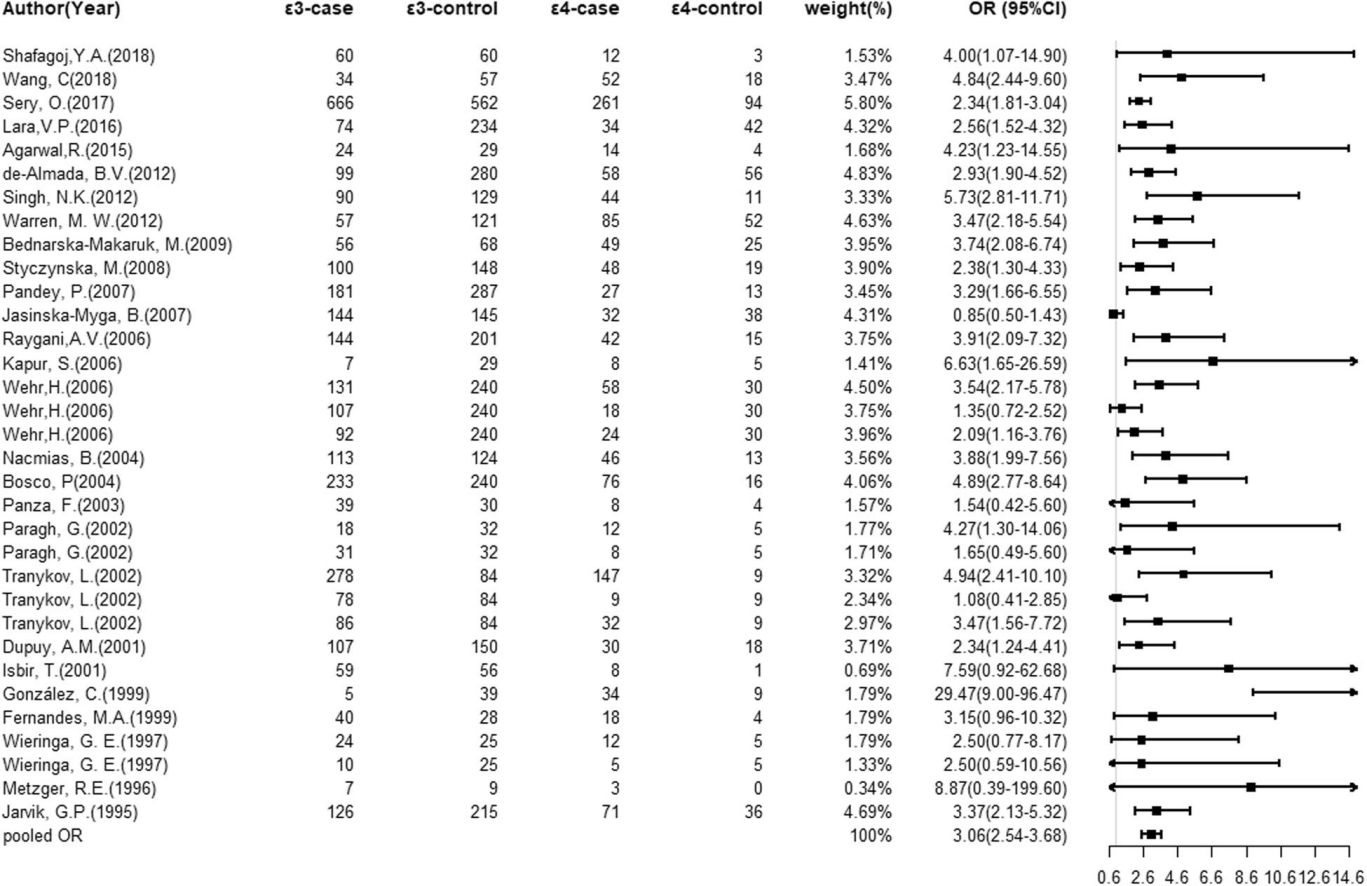
Overall comparisons of *ApoE* gene ε4 versus ε3 in association with dementia risk. Forest plots of the dementia risk associated with the *ApoE* gene ε2/ε3/ε4 alleles for ε4 vs. ε3 in all study populations.

Considering the limited numbers of *ApoE* genotypes, only allelic comparisons (ε2 versus ε3, and ε4 versus ε3) were computed. As shown in Fig. 1, carriers of ε2 allele were of a significant decrease risk for dementia (OR = 0.69; 95% CI: 0.59 to 0.81; *P* < 0.01) compared to these of ε3 allele, with a low probability of publication bias as reflected by the suggestive symmetry of the funnel plot (Supplementary Fig. S2) and the Egger’s test (*P* = 0.364). There was no evidence of heterogeneity for the comparison of ε2 with ε3 (*I*^2^ = 8.30%). In contrast, the ε4 allele was significantly associated with a 3.06-fold (OR = 3.06; 95% CI: 2.54–3.68; *P* < 0.01; Fig. 2) increased risk of developing dementia compared with the ε3 allele, accompanied by moderate heterogeneity (*I*^2^ = 55.10%; *P* < 0.01; Supplementary Fig. S3). In addition, there was no apparent publication bias (Egger’s test: *P* = 0.131), which improved the strength of this association.

### Stratified comparisons for dementia risk

In an attempt to examine the potential sources of heterogeneity between different subgroups, several subgroup analyses were conducted according to the dementia type, sample size, ethnicity or gender (Table 1).

**Table 1:** Subgroup analyses of the *ApoE* gene ε2/ε3/ε4 polymorphism with dementia risk.

		ε2 vs. ε3				ε4 vs. ε3			
Subgroup	Studies, n	OR	95% CI	*P*	*I*^2^	OR	95% CI	*P*	*I*^2^
Dementia type									
AD	23	0.70	0.58–0.84	**<0.01**	13.10%	3.62	3.03–4.32	**<0.01**	32.40%
VaD	3	0.44	0.18–1.07	0.07	20.6%	1.32	0.81–2.13	0.27	0.00%
Total sample size									
<200	19	0.70	0.49–0.99	**<0.05**	13.10%	3.25	2.19–4.83	**<0.01**	66.10%
≥200	14	0.68	0.56–0.85	**<0.05**	9.70%	3.05	2.54–3.67	**<0.01**	54.40%
Ethnicity in AD patients									
Caucasian	12	0.65	0.43–0.96	**<0.05**	36.40%	3.53	2.62–4.78	**<0.01**	56.90%
Asian	7	0.80	0.43–1.51	0.50	19.30%	4.76	3.39–6.69	**<0.01**	0.00%
Latinos	1	0.71	0.30–1.67	0.43	0.00%	2.93	1.90–4.52	**<0.01**	0.00%
North America	3	0.59	0.32–1.07	0.08	0.00%	3.45	2.49–4.78	**<0.01**	0.00%
Gender in AD patients									
Male	1	0.76	0.64–0.92	**<0.01**	100%	1.00	0.92–1.10	0.90	100%
Female	1	0.74	0.64–0.86	**<0.01**	100%	1.00	0.95–1.13	0.38	100%

Bold values were statistically significant differences for subgroup analyses (*P* < 0.05).

The summary effect estimates in stratified analyses were similar in meta-analysis including studies with sample size ≥200 (OR = 0.68, 95% CI: 0.56–0.85; *P* < 0.05) and sample size <200 (OR = 0.70, 95% CI: 0.49–0.99; *P* < 0.05) across the comparison of ε2 vs. ε3. In addition, the summary effect estimates were also comparable in meta-analysis including studies with sample size ≥200 (OR = 3.05, 95% CI: 2.54–6.67; *P* < 0.01) and sample size <200 (OR = 3.25, 95% CI: 2.19–4.83; *P* < 0.01) across the comparison of ε4 vs. ε3. In the subgroup analyses by dementia subtypes, the association of the *ApoE* gene with the risk of AD (OR = 3.62; 95% CI: 3.03–4.32; *P* < 0.01) was stronger across the comparison of ε4 vs. ε3. Carriers with the ε2 allele had a significant decreased risk in patients with AD (OR = 0.70; 95% CI: 0.58–0.84; *P* < 0.01) compared to these with the ε3 allele.

In terms of AD, carriers with the ε4 allele were of increased risk in Caucasian (OR = 3.53, 95% CI: 2.62–4.78; *P* < 0.01), Asian (OR = 4.76, 95% CI: 3.39–6.69; *P* < 0.01), Latinos (OR = 2.93, 95% CI: 1.90–4.52; *P* < 0.01) and North America (OR = 3.45, 95% CI: 2.49–4.78; *P* < 0.01), compared to carriers with the ε3 allele. There were consistently significant associations for the comparison of ε2 versus ε3 in the male (OR = 0.76; 95% CI: 0.64–0.92; *P* < 0.01) and female (OR = 0.74; 95% CI: 0.64–0.86; *P* < 0.01) in term of AD.

### Association of the *ApoE* gene alleles with circulating cholesterols levels

**Fig. 3:**
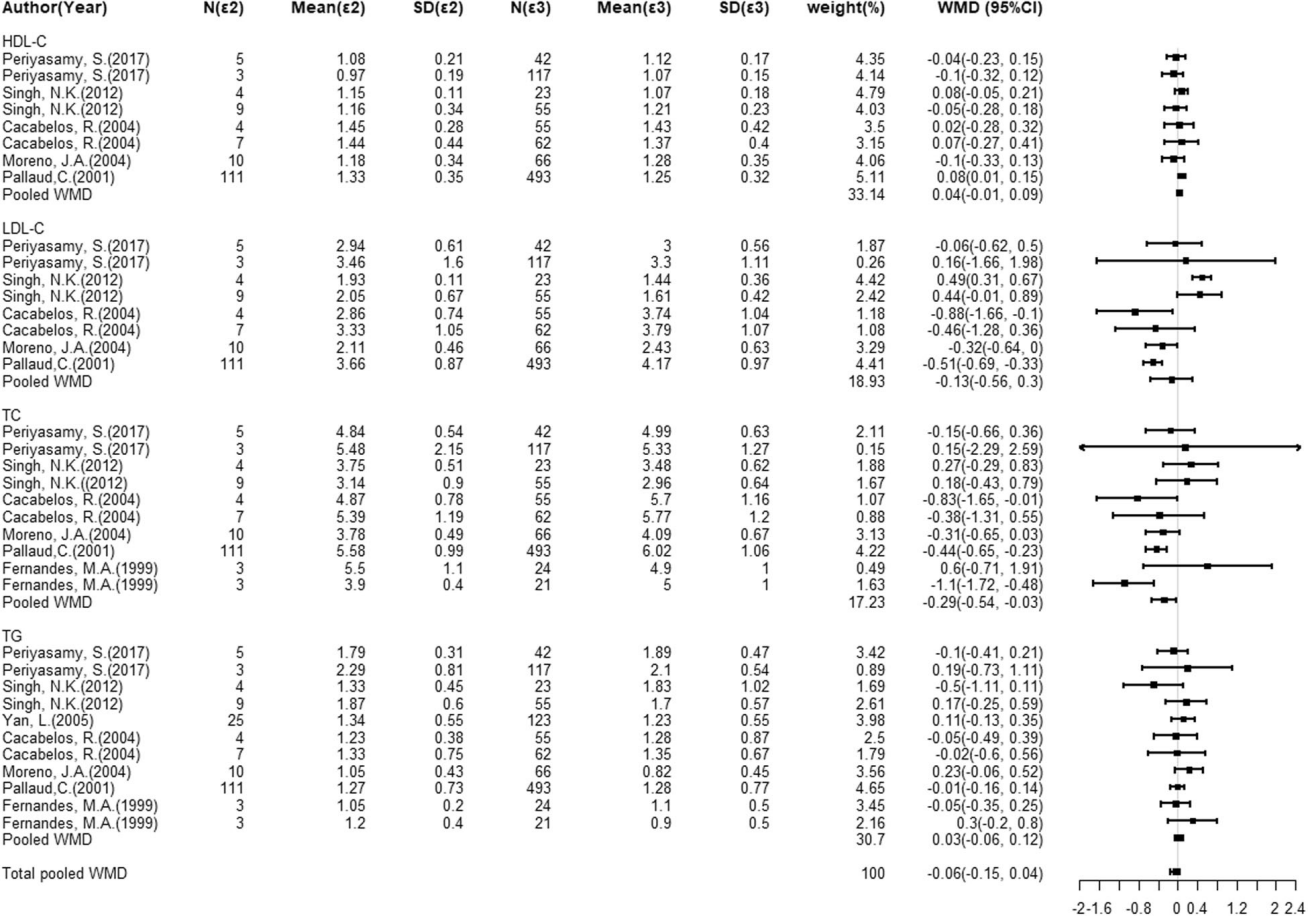
Overall circulating cholesterol levels for the comparisons of *ApoE* gene ε2 versus ε3. Forest plots of circulating cholesterol levels associated with the *ApoE* gene ε2/ε3/ε4 alleles for ε2 vs. ε3 from the available studies.

**Fig. 4:**
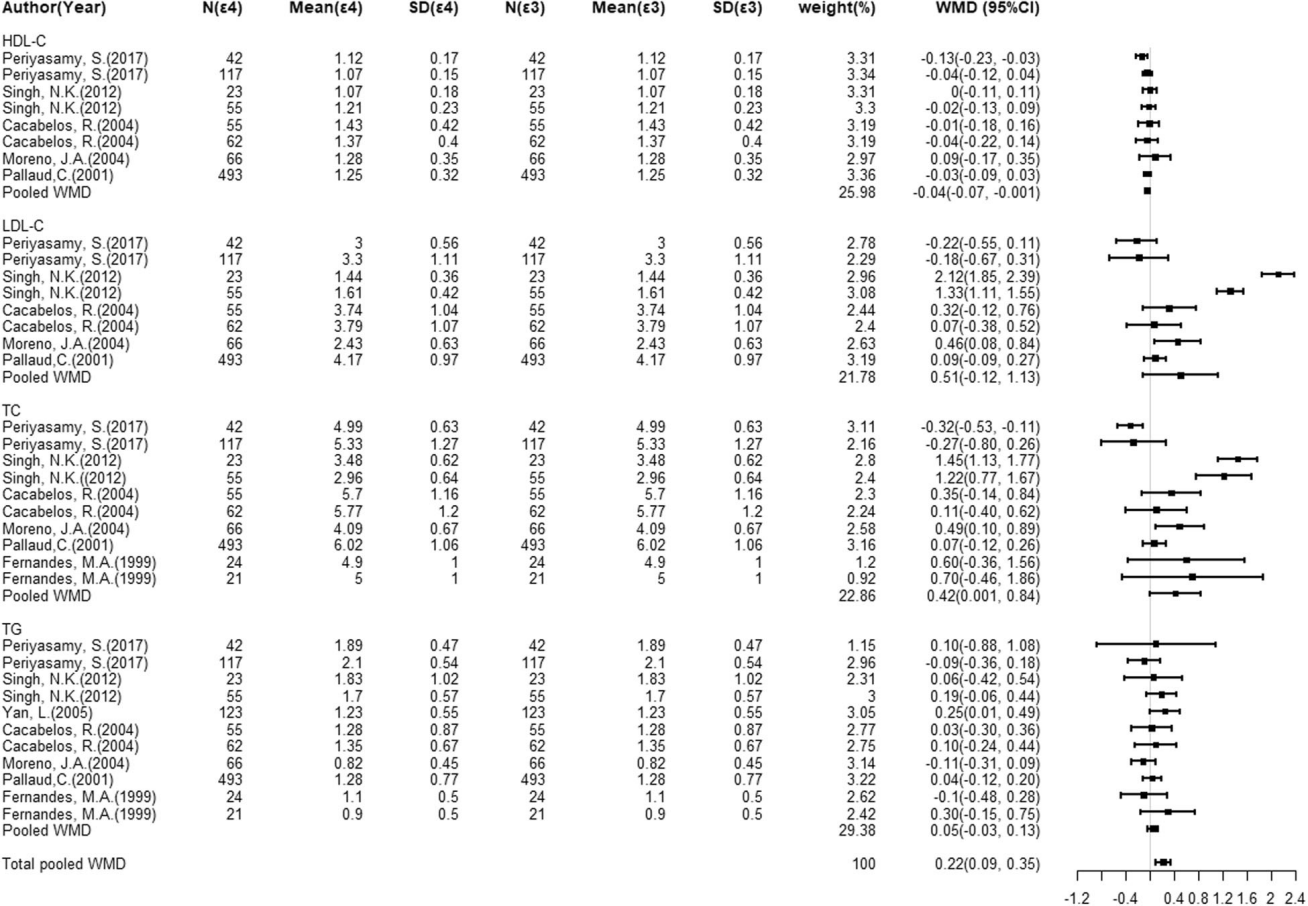
Overall circulating cholesterol levels for the comparisons of *ApoE* gene ε4 versus ε3. Forest plots of circulating cholesterol levels associated with the *ApoE* gene ε2/ε3/ε4 alleles for ε4 vs. ε3 from the available studies.

In the view of limited data on *ApoE* genotypes, mean lipid changes were only compared in ε2 vs. ε3 and ε4 vs. ε3. Figures 3 and 4 present the overall analyses of the *ApoE* gene ε2/ε3/ε4 polymorphism with circulating levels of TC, HDL-C, LDL-C, and TG under ε2 vs. ε3 and ε4 vs. ε3 comparisons. Carriers of ε2 allele had a significant reduction in circulating TC (WMD = − 0.29 mmol/L; 95% CI: −0.54 to −0.03; *P* < 0.05; Fig. 3) when compared to ε3 allele, with the moderate evidence of heterogeneity (*I*^2^ = 50%). In addition, carriers of the ε4 allele had a significant reduction in circulating HDL-C (WMD = −0.04 mmol/L; 95% CI: −0.08 to −0.001; *P* < 0.05) without heterogeneity (*I*^2^ = 0.00%), compared to carriers of the ε3 allele. As expected, higher circulating TC was observed in subjects with the ε4 allele (WMD = 0.42 mmol/l; 95% CI: 0.001–0.84; *P* < 0.05; Fig. 4) compared to the ε3 allele carriers, with a significant heterogeneity (*I*^2^ = 91.70%).

### Causal prediction of circulating lipids for dementia

According to the requirements of MR approach, the predicted OR of having AD for 1 mg/dL increment in circulating TC was 0.97 (95% CI: 0.86–0.98; *P* < 0.05) for the comparison of allele ε2 with ε3. In comparing allele ε4 with ε3, the predicted OR for a 1 mg/dL increment in TC was 1.08 (95% CI: 1.05–17.58; *P* < 0.05), while the predicted OR for a 1 mg/dL reduction in HDL-C was 2.30 (95% CI: 1.51–43.99; *P* < 0.05). This estimate was significant at a significance level of 5%, and the null hypothesis value of 1 was not included in the estimated 95% CI for the association of circulating cholesterol level and risk of dementia.

## Discussion

In this MR meta-analysis, *ApoE* gene polymorphism was utilized as an instrumental variable to evaluate the potential causal relation between circulating cholesterol and the risk of dementia. To the best of our knowledge, this is the first meta-analysis to evaluate the relationship from the perspective of the MR approach.

Cholesterol in the pathogenesis of AD remains controversial^48^. The lipid-AD associations are progressively stronger with increasing pathological certainty of an AD diagnosis. These relationships were still significant after adjustment for the *ApoE* genotype and for other known risk factors^3^. In addition, it is still unclear that this association is causal or confouder, because of the involvement of many confounding factors including age, BMI and genetic background, as well as the complex biological effects of circulating lipid levels. Therefore, the MR approach is used to assess the causal relation of circulating cholesterol levels with dementia risk.

Some evidence supported a close relation between *ApoE* genetic alterations and the circulating HDL-C and TC profiles in the meta-analyses^24,49–51^, which revealed that the *ApoE* gene ε4 allele was significantly associated with reduced circulating HDL-C and increased circulating TC levels. Previous MR meta-analyses have assessed the association between genetically circulating cholesterol levels and other diseases including cancer and hypertension. Yang et al. indicated that the predicted odds of overall cancer for a 1 mg/dL reduction in circulating HDL-C was 1.14^52^. Another study showed that a 1 mmol/L increment in TC corresponded to a 4.58-times higher likelihood of developing hypertension, and the same increase in LDL-C corresponded to a 3.97-times higher likelihood^24^. Because genotypes are invariant over time and can exert an effect on circulating cholesterol levels over a lifetime.

As for the molecular mechanisms, there has been increasing evidence in basic medical research. Umeda et al. observed that hypercholesterolemia accelerates the intraneuronal accumulation of Aβ oligomers and subsequent synapse loss, thus resulting in memory impairment in AD mouse models^53^. Chen et al. found that increased levels of cholesterol affected the function and structure of endolysosomes, leading to the deposition of Aβ and phosphorylated tau protein in the brain of AD rabbits^54^. A recent study also demonstrated that high cholesterol and 27-hydroxycholesterol levels affect memory consolidation in experimental studies, suggesting that this cholesterol metabolite could link peripheral cholesterol to AD pathogenesis^55^. In a meta-analysis, the use of statins was significantly associated with a reduced risk of all-caused dementia (adjusted RR (aRR) = 0.849, 95% CI: 0.787–0.916) and AD (aRR = 0.719, 95% CI: 0.576 to 0.899)^56^. Another meta-analysis indicated that the use of statins might benefit all AD subjects (HR = 0.80; 95% CI: 0.68–0.95) and may be most beneficial in subjects with an *ApoE* ε4/4 genotype^57^. Together, these studies strongly suggest that increased levels of circulating cholesterol play an important role in the pathogenesis of AD. Consequently, these studies suggested that increased levels of circulating cholesterol might play a causal role in the pathogenesis of AD.

Despite the strengths of this meta-analysis, some possible limitations should be acknowledged. Firstly, because we retrieved published literatures in English, selective publication bias could not be ruled out completely. Secondly, we only focused on the *ApoE* gene ε2/ε3/ε4 polymorphism as well as did not cover other candidate genes or polymorphisms in this gene, which might restrict the statistical power. Thirdly, the cholesterol levels were measured only once for almost all eligible studies which could not reflect the long-term cholesterol profiles in the development of dementia. Fourthly, this meta-analysis was conducted with the use of summarized data rather than individual participant data, with age-stratified, homozygous and heterozygous genotypes analyses unavailable. Fifthly, an essential requirement of MR was that a pleiotropic effect of the *ApoE* gene ε2/ε3/ε4 polymorphism was not be calculated and might beyond the capability to eliminate this effect in this meta-analysis. Therefore, a solid conclusion could not be produced from this meta-analysis which should be treated cautiously, until large, well-designed, prospective studies confirm our findings.

In conclusion, our results indicate that reduced circulating HDL-C and increased TC levels may be potential risk factors for dementia. Our findings in this study are promising for future practical applications.

## Supplementary information

Supplementary Fig. S1 Flow diagram of the search strategy and study selection.

Supplementary Fig. S2 Begg’s funnel plots for the comparisons of *ApoE* gene ε2 versus ε3.

Supplementary Fig. S3 Begg’s funnel plots for the comparisons of *ApoE* gene ε4 versus ε3.
