# Supplementary figures and images for: Causal association of circulating cholesterol levels with dementia: a mendelian randomization meta-analysis
Source: Transl Psychiatry. 2020 May 12;10:145. doi: 10.1038/s41398-020-0822-x (PMC7217910; doi:10.1038/s41398-020-0822-x)

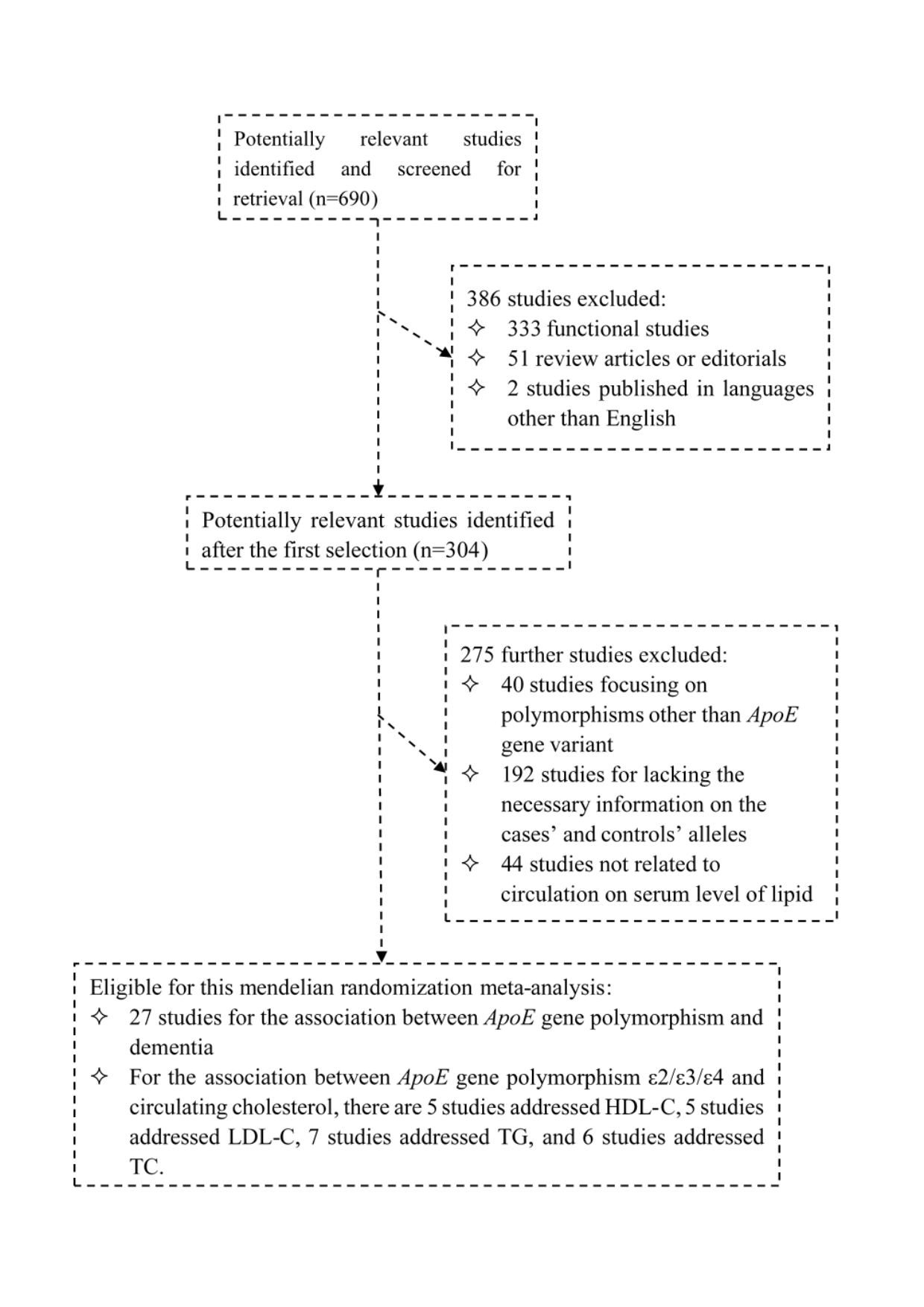

Supplement: Supplementary file 1 — Supplementary Fig. S1 Flow diagram of the search strategy and study selection. [file 41398_2020_822_MOESM1_ESM.tif]

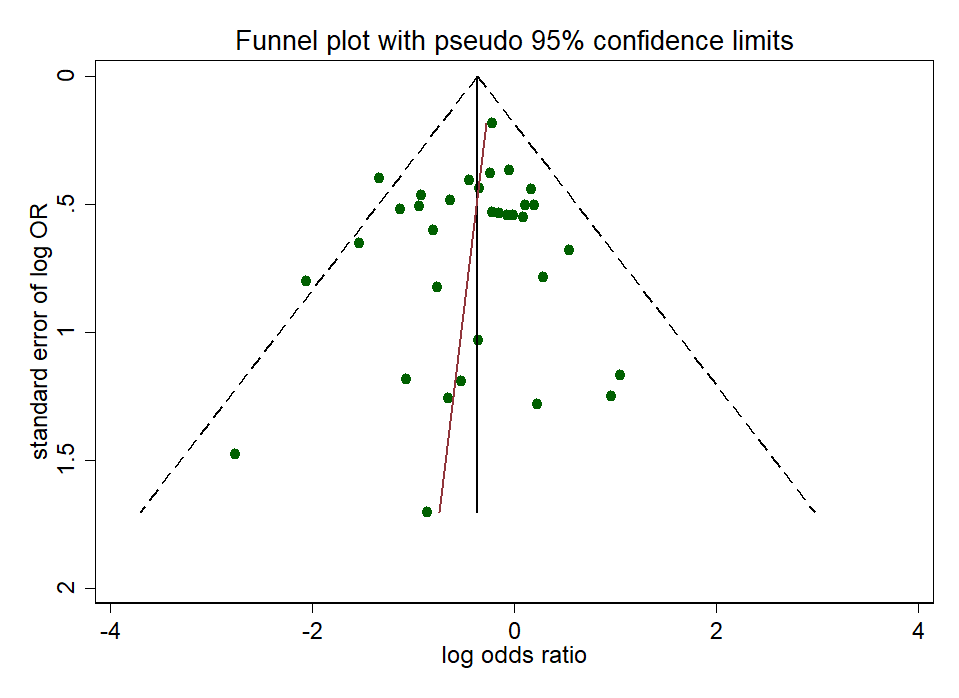

Supplement: Supplementary file 2 — Supplementary Fig. S2 Begg’s funnel plots for the comparisons of ApoE gene ε2 versus ε3. [file 41398_2020_822_MOESM2_ESM.tif]

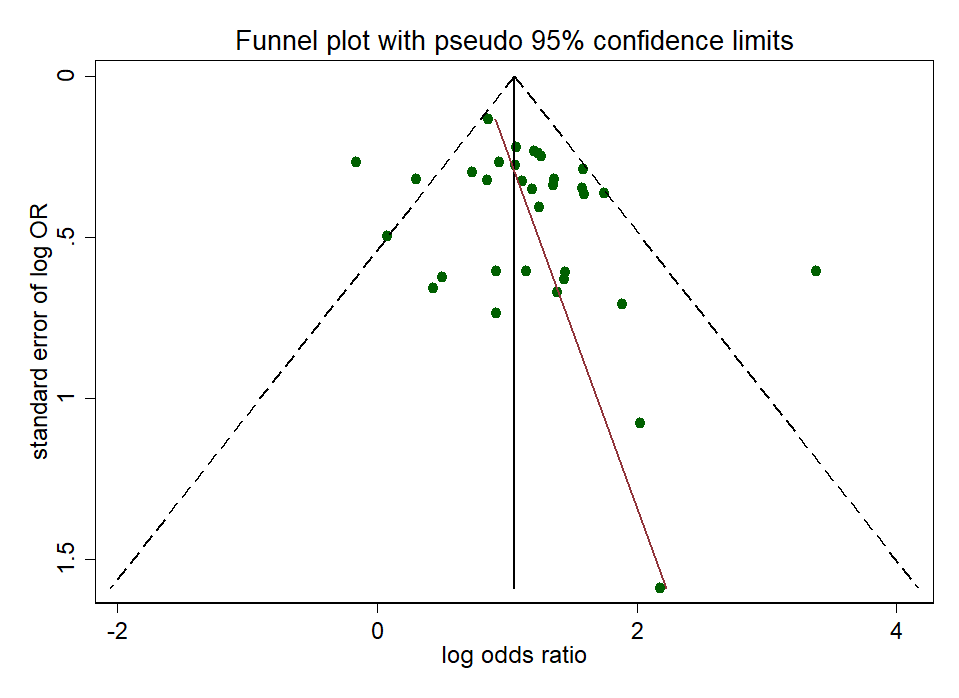

Supplement: Supplementary file 3 — Supplementary Fig. S3 Begg’s funnel plots for the comparisons of ApoE gene ε4 versus ε3. [file 41398_2020_822_MOESM3_ESM.tif]
